# Supplementary material for: Association between oral microbiome and seven types of cancers in East Asian population: a two-sample Mendelian randomization analysis
Source: Front Mol Biosci. 2023 Nov 21;10:1327893. doi: 10.3389/fmolb.2023.1327893 (PMC10702768; doi:10.3389/fmolb.2023.1327893)
Supplement: Supplementary file 1 [file DataSheet1.ZIP › Supplementary Materials/MR plots of saliva/saliva═╝/colorectal cancer/pheno.3354_to_colorectal cancer_forest.pdf]

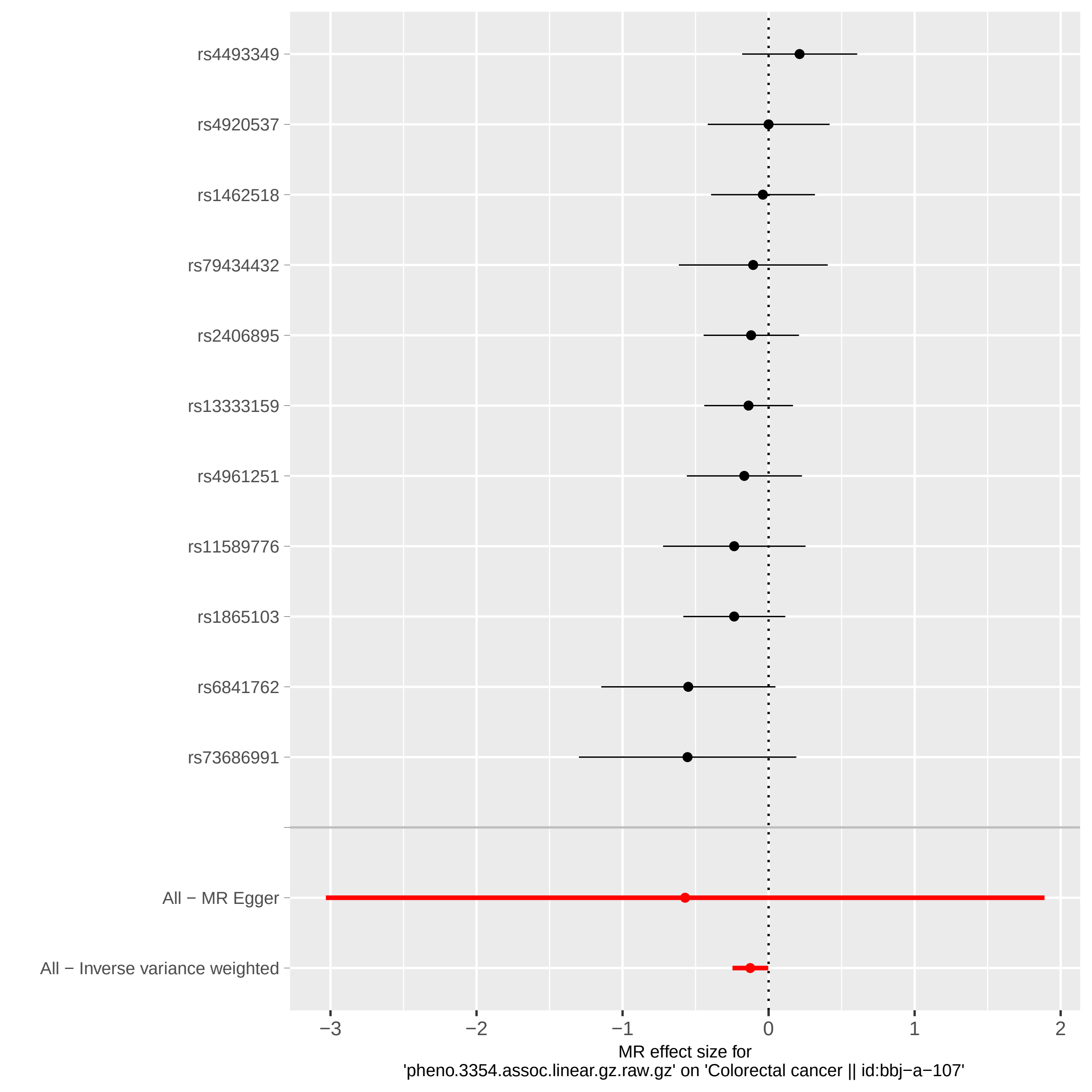

rs4493349
rs4920537
rs1462518
rs79434432
rs2406895
rs13333159
rs4961251
rs11589776
rs1865103
rs6841762
rs73686991
All − MR Egger
All − Inverse variance weighted
−3
−2
−1
0
1
2
MR effect size for
'pheno.3354.assoc.linear.gz.raw.gz' on 'Colorectal cancer || id:bbj−a−107'
